# The association of mammographic density with risk of contralateral breast cancer and change in density with treatment in the WECARE study

**DOI:** 10.1186/s13058-018-0948-4

**Published:** 2018-03-22

**Authors:** Julia A. Knight, Kristina M. Blackmore, Jing Fan, Kathleen E. Malone, Esther M. John, Charles F. Lynch, Celine M. Vachon, Leslie Bernstein, Jennifer D. Brooks, Anne S. Reiner, Xiaolin Liang, Meghan Woods, WECARE Study Collaborative Group, Marinela Capanu, Xiaolin Liang, Irene Orlow, Anne S. Reiner, Mark Robson, Meghan Woods, Leslie Bernstein, John D. Boice, Jennifer Brooks, Patrick Concannon, Dave V. Conti, David Duggan, Joanne W. Elena, Robert W. Haile, Esther M. John, Julia A. Knight, Charles F. Lynch, Kathleen E. Malone, Lene Mellemkjær, Jørgen H. Olsen, Daniela Seminara, Roy E. Shore, Marilyn Stovall, Daniel O. Stram, Marc Tischkowitz, Duncan C. Thomas, Kristina Blackmore, Anh T. Diep, Judy Goldstein, Irene Harris, Rikke Langballe, Cecilia O’Brien, Susan Smith, Rita Weathers, Michele West, Jonine L. Bernstein

**Affiliations:** 1 Lunenfeld-Tanenbaum Research Institute, Sinai Health System, 60 Murray Street Box 18, Toronto, ON M6P 2G3 Canada; 2 Dalla Lana School of Public Health, University of Toronto, Toronto, ON Canada; 3 Prevention and Cancer Control, Cancer Care Ontario, Toronto, ON Canada; 4 Fred Hutchinson Cancer Research Center, Seattle, WA USA; 5 Cancer Prevention Institute of California, Fremont, CA USA; 6 Department of Health Research and Policy (Epidemiology) and Stanford Cancer Institute, Stanford University School of Medicine, Stanford, CA USA; 7 University of Iowa, Iowa City, IA USA; 8 Mayo Clinic, Rochester, MN USA; 9 Beckman Research Institute, City of Hope National Medical Center, Duarte, CA USA; 10 Memorial Sloan Kettering Cancer Center, New York, NY USA

**Keywords:** Mammographic density, Contralateral breast cancer, Breast cancer treatment

## Abstract

**Background:**

Mammographic density (MD) is an established predictor of risk of a first breast cancer, but the relationship of MD to contralateral breast cancer (CBC) risk is not clear, including the roles of age, mammogram timing, and change with treatment. Multivariable prediction models for CBC risk are needed and MD could contribute to these.

**Methods:**

We conducted a case-control study of MD and CBC risk in phase II of the WECARE study where cases had a CBC diagnosed ≥ 2 years after first diagnosis at age <55 years and controls had unilateral breast cancer (UBC) with similar follow-up time. We retrieved film mammograms of the unaffected breast from two time points, prior to/at the time of the first diagnosis (253 CBC cases, 269 UBC controls) and ≥ 6 months up to 48 months following the first diagnosis (333 CBC cases, 377 UBC controls). Mammograms were digitized and percent MD (%MD) was measured using the thresholding program Cumulus. Odds ratios (OR) and 95% confidence intervals (CI) for association between %MD and CBC, adjusted for age, treatment, and other factors related to CBC, were estimated using logistic regression. Linear regression was used to estimate the association between treatment modality and change in %MD in 467 women with mammograms at both time points.

**Results:**

For %MD assessed following diagnosis, there was a statistically significant trend of increasing CBC with increasing %MD (*p* = 0.03). Lower density (<25%) was associated with reduced risk of CBC compared to 25 to < 50% density (OR 0.69, 95% CI 0.49, 0.98). Similar, but weaker, associations were noted for %MD measurements prior to/at diagnosis. The relationship appeared strongest in women aged < 45 years and non-existent in women aged 50 to 54 years. A decrease of ≥ 10% in %MD between first and second mammogram was associated marginally with reduced risk of CBC (OR 0.63, 95% CI 0.40, 1.01) compared to change of <10%. Both tamoxifen and chemotherapy were associated with statistically significant 3% decreases in %MD (*p* < 0.01).

**Conclusions:**

Post-diagnosis measures of %MD may be useful to include in CBC risk prediction models with consideration of age at diagnosis. Chemotherapy is associated with reductions in %MD, similar to tamoxifen.

**Electronic supplementary material:**

The online version of this article (10.1186/s13058-018-0948-4) contains supplementary material, which is available to authorized users.

## Background

Mammographic density (MD) has a well-established and strong relationship with increased risk of first primary breast cancer, with an odds ratio (OR) of 4.6 for high versus low density categories in a large meta-analysis [1]. MD is defined as the area that appears white on a mammogram in contrast to the dark non-dense or radiologically translucent area. Dense areas consist largely of glandular and stromal tissue while non-dense areas are largely adipose tissue. Percent MD (%MD) is the proportion of the total breast area on the mammogram occupied by dense tissue.

Breast cancer is the most common cancer in women worldwide [2] and survival rates are high in developed countries [3]. Thus, the population of breast cancer survivors at risk of a second primary cancer is large and increasing. In these women, the risk of developing contralateral breast cancer (CBC) is higher than the risk of developing a first breast cancer in the general population [4]. Therefore, predicting and reducing CBC risk is an important clinical consideration for breast cancer survivors. In particular, effective means of CBC risk assessment are needed for making decisions about prophylactic contralateral mastectomy.

The recommended follow up for women who have survived a first primary breast cancer includes annual screening mammography of the contralateral breast [5], making MD a feasible measure to contribute to the assessment of CBC risk. There is some evidence showing that higher MD at the time of a diagnosis of ductal carcinoma in situ (DCIS) is associated with increased CBC risk [6–8]. Two large studies including women with both DCIS and invasive cancer observed higher rates of any second breast primary [9] or CBC [10] in women with heterogeneously dense or extremely dense Breast Imaging Reporting and Data System (BI-RADS) categories before diagnosis compared to women with almost entirely fatty breasts. Similar results were observed in women with invasive breast cancer only [11]. However, another study of women with invasive breast cancer did not identify association between MD around the time of diagnosis and CBC risk [12]. No previous study has specifically assessed MD following treatment for a first primary breast cancer as a risk factor for and potential predictor of CBC. One study assessed changes in %MD between the baseline and follow-up mammograms and found a statistically significant reduction in CBC risk in women with a 10% or greater decrease in %MD compared with women who had a difference of < 10% [12]. MD generally decreases following tamoxifen use as reviewed elsewhere [13, 14] and may also decrease with chemotherapy [12, 15]. Therefore, the effect of treatment should be considered when assessing the role of MD in CBC risk. Furthermore, it is not clear whether the timing of the mammogram with respect to treatment influences the magnitude of any association with CBC risk.

We conducted a case-control study in patients with CBC (cases) and controls with unilateral breast cancer (UBC) to address the relationship between MD and CBC risk and the associations between breast cancer treatment modalities and changes in MD. We also assessed the relationship between MD and CBC risk specifically in younger women, which has not previously been assessed. Mammograms prior to or at the time of first diagnosis and mammograms obtained more than 6 months after that diagnosis were assessed for MD. The study was conducted within phase II of the Women’s Environmental Cancer and Radiation Epidemiology (WECARE) study.

## Methods

### Study population

The WECARE Study is an international population-based case-control study of women in which cases are patients with asynchronous CBC and controls are patients with UBC. The study recruitment and data collection occurred in two phases, WECARE I (2001–2004) and WECARE II (2009–2012), using similar study procedures in all centers. As the WECARE MD study was planned during WECARE II and only women recruited during this phase were asked for consent to retrieve their mammograms, only phase II is discussed further. Each study center identified eligible women through one or more population-based cancer registries. The three US study centers recruited women from registries that contribute to the NCI SEER Registry program: Seattle, Washington; Iowa; and California. Additional recruitment and data collection were conducted in Denmark and in Ontario, Canada; however, Denmark did not participate in the WECARE MD study.

WECARE study participants were diagnosed prior to age 55 years, between 1990 and 2008, with a first primary local or regional-stage invasive breast cancer. Cases were also diagnosed with a second primary invasive CBC at least 2 years later with no intervening cancer diagnosis, other than a non-melanoma skin cancer or cervical carcinoma in situ. UBC controls had no history of subsequent cancer diagnosis except for non-melanoma skin cancer or cervical carcinoma in situ up to their reference date, defined as follows. The reference date for cases was the CBC diagnosis date, while for controls it was defined by adding the interval between the first breast cancer and the CBC for the matched case to the date of breast cancer diagnosis for the control, thus matching on follow-up time. Cases and controls must also have been living in the same study reporting area during the period from first breast cancer diagnosis to the reference date. Additionally, controls must not have undergone prophylactic mastectomy of the contralateral breast. All women had to be alive at the time of contact for interview. When selected, each control was individually matched to a case on year of birth (in 5-year strata), year of diagnosis (in 4-year strata), cancer registry region, race/ethnicity, and follow-up time as described above. All study participants provided written informed consent and the study was approved by the institutional ethics review boards at the University of Iowa (IRB-01), Fred Hutchinson Cancer Research Center, Cancer Prevention Institute of California, Mount Sinai Hospital, and Memorial Sloan Kettering Cancer Center and by the Committee for the Protection of Human Subjects of the State of California.

### Data and mammogram collection

WECARE study participants were interviewed by telephone using a structured questionnaire that was designed to obtain information about events occurring before the diagnosis of the first primary breast cancer, and events that occurred during the period between diagnosis and reference date. The focus of the questionnaire was on currently known or suspected risk factors for breast cancer, including personal demographics, medical history, cancer family history, menstrual and reproductive history, hormone use, body size, cigarette smoking, and alcohol intake. Women were also asked if and where mammograms were performed before and after the first breast cancer diagnosis. Additionally, medical records including pathology reports and hospital charts were used to collect detailed treatment information (i.e., chemotherapy, hormonal therapy, and radiation therapy) for the first primary breast cancer, any recurrences experienced prior to the reference date and characteristics of the first primary tumour (e.g., estrogen receptor (ER) status, histology).

Each participating study center attempted to obtain two mammograms of the unaffected contralateral breast in each woman, one as close as possible to the period 12 months prior to diagnosis up to 1 month post diagnosis (henceforth referred to as prior to/at diagnosis) and one as close as possible to the period of > 6 months up to 18 months post diagnosis (henceforth referred to as post diagnosis). Only the cranio-caudal view of the contralateral breast was used in this study.

### Mammogram digitization and MD measurement

The film mammograms were digitized at two locations, Seattle (all US mammograms) and Toronto (Ontario mammograms), both using a Kodak Lumisys Digital Scanner. MD measurements were all done in Toronto by one experienced reader (KB) using Cumulus [16], a standard computer-assisted thresholding program used to estimate the total area and dense area of the breast from which %MD is calculated (dense area/total area × 100). Mammograms were read in batches with both mammograms from the same woman read in the same batch. Mammogram order within each batch was randomized prior to reading and the reader was blinded to case-control status and time sequence of the mammogram. Each batch included approximately the same number of cases and controls. We randomly selected 10% of each batch for repeat readings within and between batches. The Pearson correlation was 0.94 for both intra- and inter-batch repeats.

### Statistical analysis

Although the original selection of cases and controls in the WECARE study was individually matched, we were not always able to obtain mammograms from both members of a matched pair. Thus, to maximize statistical power, we used unconditional logistic regression to evaluate the relationship between %MD and CBC risk, categorizing %MD as < 25%, 25 to < 50%, and ≥ 50% in all mammograms available at each time point. These categories are based on those used in a large meta-analysis of %MD [1] with the modification of combining the two lowest and two highest categories to avoid sparse data at the extreme ends. The middle group, with %MD of 25 to < 50%, is defined as the reference category. The odds ratio (OR) and 95% confidence interval (CI) were estimated for each category. In addition, a *p* value for trend was estimated using continuous data. The %MD prior to/at diagnosis was analyzed separately from %MD post diagnosis. Before further analysis, we assessed the impact of various time window definitions prior to/at diagnosis and post diagnosis, since the mammograms obtained were often taken outside our ideal time frame. We did this by re-running a basic model with %MD categories along with three factors known to be strongly associated with %MD - age, menopausal status, and estimated body mass index (BMI) at the time of the mammogram - using different time window definitions. Menopausal status (premenopausal or postmenopausal) at the time of the mammogram was derived from reported information on menstrual status and time of last period reported during the interview and BMI at the time of the post-diagnostic mammogram was estimated from the BMI reported at first breast cancer diagnosis and at reference date, using linear interpolation. The reported BMI at first diagnosis was used in models of %MD prior to/at diagnosis. There was little variation in the OR estimates across time-window definitions (Additional file 1: Table S1). Therefore, we included any pre-diagnostic mammogram up to 3 years prior to diagnosis and we selected 4 years after diagnosis as the upper limit for post-diagnostic mammograms.

In subsequent models we also included the following factors that have previously been associated with CBC risk as well as matching factors used in the design of phase II of the WECARE study: age at first diagnosis, age at menarche, number of full-term pregnancies, first-degree family history of breast cancer, study center, race (non-Hispanic white versus other), characteristics of the first tumour (histology, stage, and ER status), and use of chemotherapy, radiation, and tamoxifen after first diagnosis. In addition to the primary analyses of %MD overall, we also considered %MD associations in subgroups defined by age at first diagnosis, menopausal status at the time of mammogram, breast cancer family history, BMI, ER status, and treatment.

We assessed whether change in %MD (defined as the difference between measurements of %MD between the two time points) was associated with CBC in the subset of women who had mammograms at both time points. In this analysis we compared women with a decrease of 10% or more with women with a change of less than 10%. Few women had an increase in %MD of 10% or more (6% of CBC cases and 7% of UBC controls) and they were excluded. This model was adjusted for age at first diagnosis, change in age, change in estimated BMI, change in menopausal status, initial %MD, number of full-term pregnancies, first-degree family history of breast cancer, study center, race (non-Hispanic white versus other), characteristics of the first tumour (histology, stage, and ER status), and use of chemotherapy, radiation, and tamoxifen after first diagnosis.

To evaluate the association between different treatment modalities and change in %MD, we used linear regression with continuous change in %MD as the dependent variable. The distribution of change in %MD was approximately normal. These models were adjusted for change in age and change in estimated BMI between the two mammograms. We examined associations with the treatment modalities (chemotherapy, radiation, and tamoxifen) individually and also in the same model with mutual adjustment. Note that other types of hormonal therapies (e.g., aromatase inhibitors) were not common in this population. We also examined common chemotherapy regimens and radiation categorized by dose (none, <1Gy, ≥1Gy). We did not adjust for case-control status as there was no association between change in MD as a continuous variable and case-control status.

## Results

We recruited 812 patients with CBC (cases) and 812 UBC controls in phase II of the WECARE study and we were able to obtain at least one mammogram in 464 patients with CBC and 500 UBC controls. Women in whom we could not obtain a mammogram in an appropriate time window (see below) were more likely to have an earlier year of first breast cancer diagnosis (65% diagnosed in 1990–1996 versus 40% in 1990–1996) and to be missing ER status (14% versus 6%), and were slightly younger (mean age 45 years versus mean age 46 years). Both groups had similar distributions of histologic type (10% and 13% lobular), stage (68% and 66% local), and, after excluding those with missing status, ER status (65% and 68% positive). There were also no differences in first-degree family history (27% and 28%). During the era of the study nearly all mammograms were film rather than digital. Given that MD assessment differs by mammogram modality, we excluded the few mammograms that were digital from the analysis (5 CBC cases and 6 UBC controls prior to/at first diagnosis, 39 CBC cases and 41 UBC controls post diagnosis); we also excluded a small number of films in which MD could not be read because of poor image quality (4 CBC cases and 4 UBC controls prior to/at first diagnosis, 11 CBC cases and 6 UBC controls post diagnosis). Based on sensitivity analyses comparing time windows as described in “Methods”, we excluded mammograms taken more than 36 months prior to or 48 months following first diagnosis. Note that only one CBC case and one UBC control mammogram occurred in the 1-month period following diagnosis and likely prior to treatment initiation. The final dataset includes mammograms from 362 CBC cases (253 with mammograms prior to/at first diagnosis and 333 with mammograms post diagnosis) and 403 UBC controls (269 with mammograms prior to/at first diagnosis and 377 post diagnosis). Among these, 467 women (224 CBC cases and 243 UBC controls) had mammograms at both time points. The median time between mammograms was 1 year.

Relevant characteristics of the CBC cases and UBC controls are shown in Table 1. Mean ages at first and second mammogram were similar in the two groups as were mean estimated BMI and distributions by center and race. Of the women who had a mammogram available prior to/at diagnosis, 73% of women with CBC and 79% of UBC controls were premenopausal. The proportion of women with CBC and UBC controls who were premenopausal and had post-diagnostic mammograms dropped to 39%.

**Table 1:** Characteristics of women with contralateral breast cancer (CBC) (cases) and unilateral breast cancer (UBC) controls

	CBC (n = 362)	UBC (n = 403)
	Mean (SD)	Mean (SD)
Age at mammogram before/at 1st diagnosis (years)	46 (6)	46 (6)
Age at mammogram after 1st diagnosis (years)	47 (6)	47 (6)
Follow-up time^a^ (years)	7.9 (3.8)	8.0 (4.0)
BMI at 1st diagnosis (kg/m^2^)	25.2 (5.5)	25.2 (5.7)
BMI^b^ at mammogram after 1st diagnosis (kg/m^2^)	25.2 (5.4)	25.8 (5.7)
Percent mammographic density before/at 1st diagnosis	37.6 (18.1)	35.8 (18.3)
Percent mammographic density after 1st diagnosis	31.4 (18.3)	28.0 (16.7)
	N (%)	N (%)
Study center		
Northern California	179 (49)	213 (53)
Seattle	82 (23)	80 (20)
Ontario	52 (14)	64 (16)
Iowa	49 (14)	46 (11)
Race		
Non-Hispanic white	295 (83)	334 (81)
Other	67 (17)	69 (19)
Histologic type at 1st diagnosis		
Non-lobular	321 (89)	353 (88)
Lobular	41 (11)	50 (12)
Stage at 1st diagnosis		
Local	251 (69)	254 (63)
Regional	106 (29)	143 (35)
Missing	5 (1)	6 (1)
Estrogen receptor at 1st diagnosis		
Positive	213 (59)	273 (68)
Negative	129 (36)	105 (26)
Missing	20 (6)	25 (6)
Chemotherapy at 1st diagnosis		
Yes	236 (65)	272 (67)
No	126 (35)	131 (33)
Radiation at 1st diagnosis		
Yes	251 (69)	179 (69)
No	111 (31)	124 (31)
Tamoxifen at 1st diagnosis		
Yes	158 (44)	221 (55)
No	204 (56)	182 (45)
Family history^c^ of breast cancer		
Yes	123 (34)	89 (22)
No	234 (65)	306 (76)
Adopted/missing	5 (1)	8 (2)
Age at menarche (years)		
<13	194 (54)	193 (48)
≥13	167 (46)	209 (52)
Missing	1 (< 1)	1 (< 1)
Full-term pregnancies		
None	101 (28)	95 (24)
1–3	241 (67)	278 (69)
≥4	20 (6)	30 (7)

*SD* standard deviation, *BMI* body mass index

^a^Time interval between first diagnosis and reference date defined as time of second diagnosis in CBC cases and time of second diagnosis in matched case for UBC controls

^b^BMI estimated from reported BMI at first diagnosis and reported BMI at reference date using interpolation

^c^First-degree family history

### Associations between %MD at two time points and CBC risk

As shown in Table 2, lower %MD measured post diagnosis was associated with reduced risk of CBC. When compared to women with density 25 to <50%, the OR for women with < 25% density was 0.69 (95% CI 0.49, 0.98). The trend for increasing risk of CBC with increasing %MD was statistically significant (*p* = 0.03). Similar, but attenuated, associations were seen for %MD measured prior to/at diagnosis. The association between %MD and CBC was most apparent in women < 45 years old (Table 3). Risk was statistically significantly lower in young women with %MD <25% post diagnosis and marginally significant prior to/at diagnosis. There was no apparent trend in women aged 50–54 years at either time point. The interaction between age and %MD was of marginal significance (*p* = 0.06) post diagnosis. When subgroups were defined by menopausal status at mammogram, the associations were somewhat stronger in premenopausal than in postmenopausal women, but the interaction *p* values were larger compared to those for interactions with age group (Additional file 1: Table S2). There was no evidence for interaction between first-degree family history of breast cancer and %MD (Table 4). None of the *p* values for interaction of %MD with BMI, ER status, or any treatment modality were statistically significant (all *p* ≥ 0.30).

**Table 2:** Association between %MD and CBC risk categories prior to/at^a^ diagnosis and post diagnosis^b^

	CBC Number (%)	UBC Number (%)	OR^c^ (95% CI)
Percent mammographic density prior to/at diagnosis			
<25%	67 (26)	81 (30)	0.77 (0.49, 1.21)
25 to <50%	125 (49)	130 (48)	1.00
≥50%	61 (24)	58 (22)	1.17 (0.73, 1.87)
*p* trend			0.12
Percent mammographic density post diagnosis			
<25%	129 (39)	181 (48)	0.69 (0.49, 0.98)
25 to <50%	152 (46)	155 (41)	1.00
≥50%	52 (16)	41 (11)	1.21 (0.74, 1.99)
*p* trend			0.03

*Abbreviations: %MD* percent mammographic density, *CBC* contralateral breast cancer, *UBC* unilateral breast cancer, *OR* odds ratio, *CI* confidence interval

^a^Pior to/at diagnosis was 36 months prior up to 1 month post diagnosis

^b^Post diagnosis was > 6 months up to 48 months post diagnosis

^c^OR adjusted for study center, race (non-Hispanic white versus other), age at mammogram, menopausal status at mammogram, estimated body mass index at mammogram, age at first diagnosis, age at menarche, number of full-term pregnancies, first-degree family history of breast cancer, histologic type, stage, estrogen receptor status of first diagnosis, chemotherapy, radiation, and tamoxifen use after first diagnosis

**Table 3:** Association of %MD categories prior to/at first diagnosis^a^ and post diagnosis^b^ with CBC risk by age

	CBC Number (%)	UBC Number (%)	OR^c^ (95% CI)
Percent mammographic density prior to/at diagnosis			
Age <45 years			
<25%	18 (20)	30 (30)	0.46 (0.20, 1.05)
25 to <50%	46 (51)	49 (49)	1.00
≥50%	26 (29)	21 (21)	2.22 (0.94, 5.25)
Age 45 to <50 years			
<25%	19 (23)	25 (27)	0.86 (0.36, 2.06)
25 to <50%	39 (48)	46 (49)	1.00
≥50%	24 (29)	22 (24)	1.22 (0.51, 2.89)
Age 50–54 years			
<25%	30 (37)	26 ((34)	0.87 (0.37, 2.04
25 to <50%	40 (49)	35 (46)	1.00
≥50%	11 (14)	15 (20)	0.60 (0.21, 1.68)
*p* interaction			0.39
Post diagnosis			
Age <45 years			
<25%	42 (34)	71 (52)	0.51 (0.27, 0.96)
25 to <50%	55 (45)	51 (37)	1.00
≥50%	26 (21)	15 (11)	1.70 (0.71, 4.05)
Age 45 to <50 years			
<25%	36 (33)	57 (45)	0.60 (0.31, 1.16)
25 to <50%	54 (49)	57 (45)	1.00
≥50%	20 (18)	13 (10)	1.70 (0.68, 4.22)
Age 50–54 years			
<25%	51 (51)	53 (47)	0.98 (0.52, 1.87)
25 to <50%	43 (43)	47 (42)	1.00
≥50%	6 (6)	13 (12)	0.51 (0.16, 1.87)
*p* interaction			0.06

*Abbreviations: %MD* percent mammographic density, *CBC* contralateral breast cancer, *UBC* unilateral breast cancer, *OR* odds ratio, *CI* confidence interval

^a^Prior to/at 1st diagnosis was 36 months prior up to 1 month post diagnosis

^b^Post diagnosis was > 6 months up to 48 months post diagnosis

^c^OR adjusted for study center, race (non-Hispanic white versus other), age at mammogram, menopausal status at mammogram, estimated body mass index at mammogram, age at first diagnosis, age at menarche, number of full-term pregnancies, first-degree family history of breast cancer, histologic type, stage, and estrogen receptor status of first diagnosis, chemotherapy, radiation, and tamoxifen use after first diagnosis

**Table 4:** Association of %MD categories prior to/at first diagnosis^a^ and post diagnosis^b^ with CBC risk by family history^c^

	CBC Number (%)	UBC Number (%)	OR^d^ (95% CI)
Percent mammographic density prior to/at diagnosis			
No family history			
<25%	44 (27)	64 (31)	0.73 (0.42, 1.27)
25 to <50%	81 (49)	97 (46)	1.00
≥50%	40 (24)	48 (23)	0.96 (0.54, 1.70)
Family history			
<25%	22 (26)	16 (29)	0.95 (0.32, 2.86)
25 to <50%	43 (51)	30 (55)	1.00
≥50%	20 (24)	9 (16)	2.19 (0.62, 7.73)
*p* interaction			0.50
Post diagnosis			
No family history			
<25%	86 (39)	132 (46)	0.76 (0.50, 1.15)
25 to <50%	102 (47)	124 (44)	1.00
≥50%	30 (14)	28 (10)	1.28 (0.69, 2.36)
Family history			
<25%	41 (37)	46 (53)	0.53 (0.25, 1.14)
25 to <50%	47 (43)	28 (33)	1.00
≥50%	22 (20)	12 (14)	1.55 (0.57, 4.22)
*p* interaction			0.59

*Abbreviations: %MD* percent mammographic density, *CBC* contralateral breast cancer, *UBC* unilateral breast cancer, *OR* odds ratio, *CI* confidence interval

^a^Prior to/at 1st diagnosis was 36 months prior up to 1 month post diagnosis

^b^Post diagnosis was > 6 months up to 48 months post diagnosis

^c^Family history is defined as having a first-degree relative (mother, sister, daughter) with breast cancer, women with unknown family history were excluded.

^d^OR adjusted for study center, race (non-Hispanic white versus other), age at mammogram, menopausal status at mammogram, estimated body mass index at mammogram, age at first diagnosis, age at menarche, number of full-term pregnancies, histologic type, stage, and estrogen receptor status of first diagnosis, chemotherapy, radiation, and tamoxifen use after first diagnosis

### Association between change in %MD and CBC risk

Compared to women with a difference of < 10% in %MD between the two mammograms, women who had a decrease of ≥ 10% had reduced risk of CBC that was of borderline statistical significance (Table 5).

**Table 5:** Association of change^a^ in %MD between prior to/at first diagnosis^b^ and post diagnosis^c^ with CBC risk

Difference in %MD	CBC^d^ Number (%)	UBC^d^ Number (%)	OR^e^ (95% CI)
Difference <10%	150 (71)	144 (64)	1.00
Decrease ≥10%	60 (29)	81 (36)	0.63 (0.40, 1.01)

*Abbreviations: %MD* percent mammographic density, *CBC* contralateral breast cancer, *UBC* unilateral breast cancer, *OR* odds ratio, *CI* confidence interval

^a^Defined as the difference between the two time points

^b^Prior to/at 1st diagnosis was 36 months prior up to 1 month post diagnosis

^c^Post diagnosis was > 6 months up to 48 months post diagnosis

^d^Excluding those with missing menopausal status information and those with an increase ≥10%

^e^OR adjusted for change in age, estimated body mass index, and menopausal status between prior to/at first diagnosis and post-diagnosis mammograms and for initial %MD, study center, race (non-Hispanic white versus other), age at first diagnosis, age at menarche, number of full-term pregnancies, histologic type, stage, and estrogen receptor status of first diagnosis, chemotherapy, radiation, and tamoxifen use after first diagnosis

### Change in %MD with treatment

Chemotherapy and tamoxifen were each associated with a statistically significant reduction in %MD (Table 6). In a mutually adjusted model, the estimated effect of each of these treatment modalities was similar (a reduction in MD of 3%), and both remained highly statistically significant (*p* = 0.006 and *p* = 0.008, respectively). Examination of chemotherapy categorized by common regimens did not yield insights into the relevance of specific types of chemotherapy (Additional file 1: Table S3). Change in %MD was not associated with radiation therapy categorized as any versus none (Table 6) or by dose categories (Additional file 1: Table S3). There was no statistical evidence of interaction between radiation and either chemotherapy or tamoxifen.

**Table 6:** Association between treatment and change^a^ in %MD

		Adjusted for age and BMI^b^			Adjusted for age, BMI^b^, and other treatments		
	Number	Estimated %MD change^c^	95% CI	*p*	Estimated %MD change^c^	95% CI	*p*
Radiation							
No	142	Reference			Reference		
Yes	325	0.2	−2.1, 2.5	0.89	0.7	−1.6, 3.0	0.57
Chemotherapy							
No	161	Reference			Reference		
Yes	306	−2.9	−5.1, −0.7	0.01	−3.0	−5.2, −0.8	0.008
Tamoxifen							
No	238	Reference			Reference		
Yes	229	−2.8	−4.9, −0.7	0.009	−3.0	−5.1, −0.9	0.006

*Abbreviations: %MD* percent mammographic density, *BMI* body mass index, *CI* confidence interval

^a^Between prior to/at first diagnosis (36 months prior up to 1 month post diagnosis) and post diagnosis (more than 6 and up to 48 months post diagnosis)

^b^Estimated BMI at time of mammogram

^c^Estimated change in %MD (post diagnosis minus prior to/at diagnosis) associated with each treatment modality

## Discussion

In women younger than 55 years at first breast cancer diagnosis the risk of CBC increased linearly with increasing %MD. The linear association was statistically significant only for %MD in the post-diagnosis period, although the magnitudes of the risk estimates were similar for both time periods. The association with post-diagnosis %MD may be age-dependent, with stronger associations for women < 45 years of age and no evidence of association for women aged 50 to 54 years at first diagnosis; the resulting interaction was marginally significant (*p* = 0.06). There was also evidence of reduced CBC risk in women with a decrease in %MD of 10% or more between the prior to/at diagnosis and the post-diagnosis mammogram compared to those with a change of less than 10%. Chemotherapy was associated with a decrease in %MD similar to the expected decrease associated with tamoxifen. Radiation was not associated with change in %MD. Treatment and other factors such as ER status are known to be associated with the risk of CBC [17, 18]. Our results suggest that the addition of %MD, which is associated with CBC risk independently of treatment, could improve the prediction of CBC risk, but factors such as the timing of the mammogram (pre-treatment versus post-treatment) and the age of the woman should be considered in future studies and CBC risk-prediction models. Prediction models can help in making decisions about prophylactic surgery of the contralateral breast after a first breast cancer diagnosis.

The %MD is well-established as a risk factor for a first primary breast cancer diagnosis [1]. Adding %MD to first primary breast cancer risk prediction models has been shown to improve risk prediction, but only slightly, with increases in C-statistics of 0.01 to 0.06, reviewed in [19] and consistent with a mammographic density-based model compared to the Gail model [20]. Fewer studies have examined the relationship between %MD and CBC risk, and they have generally focused on the relationship between %MD at or prior to diagnosis with CBC risk. There is some evidence that higher MD at the time of a DCIS diagnosis is associated with increased risk of DCIS or invasive cancer in the contralateral breast [6–8]. Similarly, increased risks of any second breast primary or CBC associated with %MD have been observed among women with one DCIS or invasive breast cancer diagnosis [9–11]. In a study similar to ours, but comparing MD only at first diagnosis in CBC cases versus UBC controls, MD was unrelated to CBC risk, although the women included in that study were older than the women in our study [12]. Thus, overall there is some, but not entirely consistent evidence of an association between %MD at or prior to diagnosis and CBC risk.

Few studies have considered the relationship between MD following a first breast cancer diagnosis and CBC risk. Only one previous study has examined change in breast density following breast cancer diagnosis and risk of CBC. This study, which matched on treatment modalities, observed that a decrease of 10% or more in MD (%MD or dense area) between first breast cancer diagnosis and a follow-up mammogram (mostly within 2 years) was associated with a statistically significant reduced risk of CBC [12]. Similar to this study, we observed that women with a decrease of 10% or more following breast cancer diagnosis had a reduced risk of CBC after adjustment for multiple variables including treatment, although the relationship was of borderline statistical significance. The consistency of the two similar studies suggests that change in %MD could be a useful metric in prediction models of CBC and/or that MD measured after treatment may be a better predictor of CBC risk than MD prior to/at diagnosis, or at least a useful addition to models. Larger studies comparing all three metrics would be useful.

We found a stronger association of %MD with CBC risk in younger women, particularly in those < 45 years of age at first breast cancer diagnosis. There is no evidence that the relationship between MD and the risk of a first breast cancer varies by age when younger women are defined as premenopausal, age <50 years, or age <55 years [1, 21], but it is not clear whether there are differences at younger ages. The %MD generally decreases with increasing age and during the transition to postmenopausal status. As there were relatively few women in the higher %MD categories at older ages and also following breast cancer treatment in our study, we likely lacked statistical power to detect significant association between %MD and CBC risk in women aged 45 –54 years. The %MD may be a stronger predictor of CBC risk in younger women, but this finding needs confirmation.

Tamoxifen has been associated with a decrease in MD in a number of studies [13, 14] and it has been suggested that the extent of decrease in MD after tamoxifen initiation may be an indicator of response to treatment and may be related to risk of breast-cancer-related endpoints [22]. Most of the change in MD that occurs with tamoxifen use happens within the first year of use [23].

To our knowledge only two prior studies have assessed changes in breast density following chemotherapy [12, 15]. In the study of Chen et al., where this was a focus, breast density was assessed using magnetic resonance imaging in women receiving neoadjuvant chemotherapy with doxorubicin and cyclophosphamide with or without taxane. The decrease in density was statistically significant following either chemotherapy regimen [15]. More recently, Sandberg et al. have also shown a significant decrease in %MD following any chemotherapy with or without other therapies [12]. These results are consistent with our observed statistically significant reduction in %MD with any chemotherapy. The decreased density with chemotherapy may occur as a result of suppression of the ovary by chemotherapy. No previous studies have assessed whether radiation exposure has any direct effect on MD or any moderating influence on the effect of other treatment modalities.

The strength of our study is that we have detailed medical record information for our cases and controls, including tumour characteristics and treatment information, as well as other self-reported information relevant to CBC risk such as family history of breast cancer. To our knowledge, our study is the first to examine differences in associations with MD both prior to/at diagnosis and post diagnosis. Only one prior study evaluated MD both before and after treatment for a first breast primary breast cancer, but did not assess the relationship between post-diagnosis MD and CBC. Our main limitation is sample size, as we had to retrieve mammograms from some time in the past and they were frequently unavailable, particularly the earlier mammograms. Therefore, we cannot assess whether %MD from post-diagnostic mammograms was more strongly associated with risk than %MD from mammograms prior to/at diagnosis. Mammogram quality was variable and this likely attenuated our results, although the use of %MD categories should reduce the misclassification. We also could not adjust for BMI assessed at the time of the mammogram, although we were able to estimate this from BMI at two other time points. Most women in this study had their initial breast cancer diagnosis during the film mammogram era; however, several studies have demonstrated that various measures of MD from digital mammograms are also strongly predictive of breast cancer risk [24–26]. Thus, our results should still be relevant. Also, our study time period and age distribution means that few women received aromatase inhibitors. Currently there is no consistent evidence showing that aromatase inhibitors reduce MD [13, 14], although they likely reduce the risk of CBC [27, 28].

## Conclusions

CBC remains a major concern for women diagnosed with a first breast cancer, but risk is variable and effective risk prediction models are needed. Such models would be essential in making decisions about prophylactic contralateral mastectomy [10]. Given that annual mammography to screen for new breast primaries in the contralateral breast is recommended for women with a first breast cancer diagnosis [5], it is reasonable to consider MD for inclusion in CBC prediction models. Our results suggest that post-diagnosis measures of %MD may be useful to consider in CBC risk prediction models. Also, %MD may be more predictive in women aged less than 50 years, although further research is needed to confirm differences by age. Change in %MD between before and after treatment should also be considered, given the potential treatment effects on MD. Further work is needed to develop and validate clinically useful CBC risk prediction models considering comprehensive information (%MD at different time points, tumour characteristics, treatment, self-reported risk factors, genetics, etc.) for inclusion and possible variation by menopausal status or age.

## Additional file

Additional file 1: **Table S1.** Comparison of odds ratios and 95% confidence intervals for association between %MD and CBC across different time windows. **Table S2.** Association of %MD categories prior to/at first diagnosis and post-diagnosis with CBC risk by menopausal status. **Table S3.** Association of chemotherapy regimen and radiation dose with change in %MD. (DOCX 19 kb)

## Abbreviations

%MD: Percent mammographic density
BMI: Body mass index
CBC: Contralateral breast cancer
CI: Confidence interval
DCIS: Ductal carcinoma in situ
ER: Estrogen receptor
MD: Mammographic density
OR: Odds ratio
UBC: Unilateral breast cancer
WECARE Study: Women’s Environmental Cancer and Radiation Epidemiology Study
