# Supplementary material for: The association of mammographic density with risk of contralateral breast cancer and change in density with treatment in the WECARE study
Source: Breast Cancer Res. 2018 Mar 22;20:23. doi: 10.1186/s13058-018-0948-4 (PMC5863854; doi:10.1186/s13058-018-0948-4)
Supplement: Supplementary file 1 — Table S1. Comparison of odds ratios and 95% confidence intervals for association between %MD and CBC across different time windows. Table S2. Association of %MD categories prior to/at first diagnosis and post-diagnosis with CBC risk by menopausal status. Table S3. Association of chemotherapy regimen and radiation dose with change in %MD. (DOCX 19 kb) [file 13058_2018_948_MOESM1_ESM.docx]

**Table S1:** Comparison of odds ratios^a^ and 95% confidence intervals for association between %MD and CBC across different time windows

| Time window in months^b^ | <25% MD | 25-<50% MD | ≥50% MD | P-trend | CBC  N | UBC  N |
| --- | --- | --- | --- | --- | --- | --- |
| Prior to/at diagnosis |  |  |  |  |  |  |
| -12 to +1 | 1.00 | 1.20 (0.77, 1.86) | 1.17 (0.67, 2.03) | 0.56 | 239 | 254 |
| -18 to +1 | 1.00 | 1.16 (0.75, 1.79) | 1.23 (0.71, 2.12) | 0.45 | 247 | 260 |
| -24 to +1 | 1.00 | 1.20 (0.78, 1.84) | 1.25 (0.72, 2.15) | 0.41 | 251 | 263 |
| -30 to +1 | 1.00 | 1.19 (0.77, 1.83) | 1.26 (0.73, 2.17) | 0.39 | 251 | 265 |
| -36 to +1 | 1.00 | 1.22 (0.79, 1.87) | 1.32 (0.77, 2.27) | 0.30 | 253 | 269 |
| >-36 to +1 | 1.00 | 1.25 (0.81, 1.91) | 1.36 (0.80, 2.32) | 0.25 | 257 | 271 |
|  |  |  |  |  |  |  |
| Post-diagnosis |  |  |  |  |  |  |
| +6 to +18 | 1.00 | 1.24 (0.84, 1.82) | 1.64 (0.94, 2.87) | 0.08 | 248 | 292 |
| +6 to +24 | 1.00 | 1.28 (0.89, 1.84) | 1.72 (1.01, 2.93) | 0.04 | 279 | 327 |
| +6 to +30 | 1.00 | 1.23 (0.87, 1.74) | 1.62 (0.96, 2.73) | 0.06 | 300 | 345 |
| +6 to +36 | 1.00 | 1.28 (0.91, 1.80) | 1.66 (1.00, 2.75) | 0.04 | 320 | 358 |
| +6 to +48 | 1.00 | 1.35 (0.97, 1.88) | 1.71 (1.04, 2.82) | 0.02 | 333 | 377 |
| +6 to >+48 | 1.00 | 1.29 (0.95, 1.75) | 1.65 (1.04, 2.63) | 0.02 | 408 | 449 |

^a^Adjusted for age, menopausal status and estimated BMI at mammogram

^b^Months relative to first diagnosis

**Table S2:** Association of %MD categories prior to/at first diagnosis^a^ and post-diagnosis^b^ with CBC risk by menopausal status^c^

|  | CBC  N (%) | UBC  N (%) | OR^d^ (95% CI^e^) |
| --- | --- | --- | --- |
| **Prior to/at diagnosis** |  |  |  |
| Premenopause |  |  |  |
| <25% | 42 (23) | 60 (28) | 1.00 |
| ≥25% | 142 (77) | 151 (72) | 1.53 (0.91, 2.59) |
| Postmenopause |  |  |  |
| <25% | 25 (37) | 21 (37) | 1.00 |
| ≥25% | 43 (63) | 36 (63) | 0.59 (0.20, 1.79) |
| P-interaction |  |  | 0.44 |
|  |  |  |  |
| **Post-diagnosis** |  |  |  |
| Premenopause |  |  |  |
| <25% | 35 (27) | 67 (46) | 1.00 |
| ≥25% | 95 (73) | 80 (54) | 2.26 (1.24, 4.11) |
| Postmenopause |  |  |  |
| <25% | 94 (47) | 113 (50) | 1.00 |
| ≥25% | 108 (53) | 115 (50) | 1.30 (0.85, 2.01) |
| P-interaction |  |  | 0.04 |

^a^36 months prior up to one month post-diagnosis

^b^>6 months up to 48 months post-diagnosis

^c^At the time of the mammogram, excluding women for whom menopausal status could not be determined

^d^Odds ratio adjusted for study centre, race (non-Hispanic white versus other), age at mammogram, menopausal status at mammogram, estimated BMI at mammogram, age at first diagnosis, age at menarche, number of full-term pregnancies, histology, stage, and ER status of first diagnosis, chemotherapy, radiation, and tamoxifen use after first diagnosis

^e^Confidence interval

**Table S3:** Association of chemotherapy regimen and radiation dose with change^a^ in %MD

|  | CBC  N (%) | UBC  N (%) | Estimated %MD change^b^ | 95% CI^c^ | P |
| --- | --- | --- | --- | --- | --- |
| Chemotherapy |  |  |  |  |  |
| None | 84 | 77 | Reference |  |  |
| With anthracyclines | 58 | 71 | -2.7 | -5.4, 0.0 | 0.05 |
| Other regimens | 82 | 95 | -3.1 | -5.6, -0.6 | 0.02 |
| Radiation |  |  |  |  |  |
| None | 66 | 76 | Reference |  |  |
| <1 Gray | 77 | 66 | 0.6 | -2.1, 3.3 | 0.68 |
| ≥1 Gray | 81 | 101 | -0.6 | -3.2, 1.9 | 0.62 |

^a^Between prior to/at first diagnosis (36 months prior up to one month post-diagnosis) and post-diagnosis (more than 6 and up to 48 months post-diagnosis)

^b^Estimated change in %MD (post-diagnosis minus prior to/at diagnosis) associated with each chemotherapy regimen or radiation dose adjusted for age and estimated BMI at each mammogram

^c^Confidence interval
